# Association Between Responsibility for the Death of Others and Postdeployment Mental Health and Functioning in US Soldiers

**DOI:** 10.1001/jamanetworkopen.2021.30810

**Published:** 2021-11-01

**Authors:** Amanda J. Khan, Laura Campbell-Sills, Xiaoying Sun, Ronald C. Kessler, Amy B. Adler, Sonia Jain, Robert J. Ursano, Murray B. Stein

**Affiliations:** 1 Department of Psychiatry, University of California San Diego, La Jolla; 2 Herbert Wertheim School of Public Health, University of California San Diego, La Jolla; 3 Department of Health Care Policy, Harvard Medical School, Boston, Massachusetts; 4 Walter Reed Army Institute of Research, Silver Spring, Maryland; 5 Center for the Study of Traumatic Stress, Department of Psychiatry, Uniformed Services University of the Health Sciences, Bethesda, Maryland; 6 Psychiatry Service, Veterans Affairs San Diego Healthcare System, San Diego, California

## Abstract

**Importance:**

Rates of suicidal thoughts and behaviors (STBs) in US soldiers have increased sharply since the terrorist attacks on September 11, 2001, and postdeployment posttraumatic stress disorder (PTSD) remains a concern. Studies show that soldiers with greater combat exposure are at an increased risk for adverse mental health outcomes, but little research has been conducted on the specific exposure of responsibility for the death of others.

**Objective:**

To examine the association between responsibility for the death of others in combat and mental health outcomes among active-duty US Army personnel at 2 to 3 months and 8 to 9 months postdeployment.

**Design, Setting, and Participants:**

This cohort study obtained data from a prospective 4-wave survey study of 3 US Army brigade combat teams that deployed to Afghanistan in 2012. The sample was restricted to soldiers with data at all 4 waves (1-2 months predeployment, and 2-3 weeks, 2-3 months, and 8-9 months postdeployment). Data analysis was performed from December 12, 2020, to April 23, 2021.

**Main Outcomes and Measures:**

Primary outcomes were past-30-day PTSD, major depressive episode, STBs, and functional impairment at 2 to 3 vs 8 to 9 months postdeployment. Combat exposures were assessed using a combat stress scale. The association of responsibility for the death of others during combat was tested using separate multivariable logistic regression models per outcome adjusted for age, sex, race and ethnicity, marital status, brigade combat team, predeployment lifetime internalizing and externalizing disorders, and combat stress severity.

**Results:**

A total of 4645 US soldiers (mean [SD] age, 26.27 [6.07] years; 4358 men [94.0%]) were included in this study. After returning from Afghanistan, 22.8% of soldiers (n = 1057) reported responsibility for the death of others in combat. This responsibility was not associated with any outcome at 2 to 3 months postdeployment (PTSD odds ratio [OR]: 1.23 [95% CI, 0.93-1.63]; *P* = .14; STB OR: 1.19 [95% CI, 0.84-1.68]; *P* = .33; major depressive episode OR: 1.03 [95% CI, 0.73-1.45]; *P* = .87; and functional impairment OR: 1.12 [95% CI, 0.94-1.34]; *P* = .19). However, responsibility was associated with increased risk for PTSD (OR, 1.42; 95% CI, 1.09-1.86; *P* = .01) and STBs (OR, 1.55; 95% CI, 1.03-2.33; *P* = .04) at 8 to 9 months postdeployment. Responsibility was not associated with major depressive episode (OR, 1.30; 95% CI, 0.93-1.81; *P* = .13) or functional impairment (OR, 1.13; 95% CI, 0.94-1.36; *P* = .19). When examining enemy combatant death only, the pattern of results was unchanged for PTSD (OR, 1.44; 95 CI%, 1.10-1.90; *P* = .009) and attenuated for STBs (OR, 1.46; 95 CI%, 0.97- 2.20; *P* = .07).

**Conclusions and Relevance:**

This cohort study found an association between being responsible for the death of others in combat and PTSD and STB at 8 to 9 months, but not 2 to 3 months, postdeployment in active-duty soldiers. The results suggest that delivering early intervention to those who report such responsibility may mitigate the subsequent occurrence of PTSD and STBs.

## Key Points

**Question:**

Is responsibility for the death of others during combat associated with postdeployment mental health problems in active-duty soldiers?

**Findings:**

In this cohort study of 4645 US soldiers who deployed to Afghanistan, responsibility for the death of others during combat as reported by soldiers was associated with greater risk of posttraumatic stress disorder (PTSD) and suicidal thoughts and behaviors (STBs) at 8 to 9 months, but not 2 to 3 months, postdeployment and was not associated with the risk of a major depressive episode or functional impairment at any time point.

**Meaning:**

The findings suggest that a critical time window exists after redeployment to provide interventions to prevent PTSD and STBs among active-duty soldiers who reported responsibility for the death of others during combat.

## Teaser

This cohort study examines the risk of posttraumatic stress disorder, suicidality, depression, and functional impairment in US Army service members who were exposed to combat experiences.

## Introduction

The association between deployment experiences and postdeployment mental health outcomes in military personnel has been extensively studied.^1,2,3^ Combat exposure is associated with postdeployment posttraumatic stress disorder (PTSD), depression, and suicidal thoughts and behaviors (STBs).^4,5,6,7,8,9^ Consistent with fear-based conceptualizations of trauma response,^10^ most studies examining combat have focused on the impact of threats to one’s life (eg, taking enemy fire) or witnessing harm done to others.^11,12,13,14,15,16^ However, evidence has shown that other specific combat experiences may confer greater risk.^6,17,18,19^

In studies of combat-deployed military personnel, many soldiers report being responsible for death and violence, but little attention has been paid to the long-term outcomes of such a traumatic event.^2,20,21,22^ The current diagnosis of PTSD does not explicitly identify participation in harming or killing others as meeting criterion A of the *DSM-5* (*Diagnostic and Statistical Manual of Mental Disorders *[Fifth Edition]) PTSD diagnosis.^20,22,23^ However, despite soldiers’ preparation and training to use violence, being responsible for someone else’s death may adversely affect their mental health, in which case screenings and interventions could be developed to mitigate these outcomes.^20,21,24,25^ Most evidence of mental health sequelae is found in studies of veterans. Across multiple war eras (Vietnam, Gulf, and Iraq/Afghanistan), being responsible for the death of others has been associated with a PTSD diagnosis and the most severe PTSD symptoms.^21,26,27,28,29^ Furthermore, this association persists after adjusting for other combat exposures. Being responsible for the death of others during combat has also been associated with STBs, demonstrating large effect sizes compared with other types of combat experiences.^17,18,28,30^ However, whether these findings extend to active-duty soldiers in the post–September 11, 2001 era,^31,32,33^ a group whose suicide rates have increased substantially over the past decade, remains relatively unexplored.^34,35^

In the current study, we examined the association between responsibility for the death of others in combat and postdeployment mental health outcomes (eg, PTSD, major depressive episode [MDE], STBs, and functional impairment) among active-duty US Army personnel. Because delayed onset of mental health problems may occur after deployment,^36^ this study assessed outcomes at both 2 to 3 months and 8 to 9 months postdeployment.

## Methods

Data for this cohort study were obtained from the Pre/Post Deployment Study of the Army Study to Assess Risk and Resilience in Servicemembers (Army STARRS),^37,38^ a prospective, multiwave panel survey of 3 US Army brigade combat teams (BCTs) that deployed to Afghanistan in 2012 for approximately 10 months on average. Baseline evaluation occurred 1 to 2 months predeployment. Follow-up assessments were conducted at approximately 2 to 3 weeks, 2 to 3 months, and 8 to 9 months postdeployment. Surveys were conducted at the BCTs’ home posts, except the 8- to 9-month postdeployment survey, which was conducted online or by telephone. Respondents provided written informed consent for participation in the surveys. Survey procedures were approved by the Human Subjects Committees at the collaborating institutions (including the Uniformed Services University of the Health Sciences for the Henry M. Jackson Foundation; the Institute for Social Research at the University of Michigan, Ann Arbor; Harvard Medical School; and University of California San Diego, La Jolla). We followed the Strengthening the Reporting of Observational Studies in Epidemiology (STROBE) reporting guideline.

At the predeployment baseline, 9949 soldiers were present for duty in the 3 BCTs. Of these soldiers, 9488 consented to participate, and 8558 completed the baseline survey and consented to link their survey responses to their US Army or Department of Defense administrative records. Approximately 90% (n = 7742) of this eligible baseline sample deployed to Afghanistan. The sample for the current study was restricted to deployed soldiers with data across all 4 waves of the survey (n = 4645).

### Measures

#### Combat Stress

A previous report described a combat stress scale,^39^ which consisted of Pre/Post Deployment Study survey items to assess the frequency of exposure to 10 combat experiences. The item-level scoring^40^ recoded the original frequency ratings (never, 1 time, 2-4 times, 5-9 times, or ≥10 times) to 0/1 or 0/1/2. For this study, the combat stress scale was modified to remove 3 items that assessed responsibility for the deaths of enemy combatants, noncombatants, or US personnel or allies. The remaining 7 items assessed going on combat patrols, firing rounds or taking enemy fire, being wounded, having a close call, having unit members seriously wounded or killed, witnessing homes or villages destroyed, and seeing severely wounded or dying or dead people. Total scores on the modified combat stress scale ranged from 0 to 8, with higher scores reflecting greater severity.

The combat stress scale was administered to all soldiers within 2 to 3 weeks of returning from the index deployment. Almost half of the sample had deployed previously (2228 of 4645 [48.0%]); for those soldiers, combat stress during past deployments was measured in the predeployment survey. The assessment varied according to number of and time since previous deployments. Those with only 1 past deployment responded in reference to that experience. Those with multiple past deployments responded in reference to (1) the most recent deployment if it occurred within the past year or (2) deployments collapsed across all previous experiences if all occurred more than 1 year ago.

#### Functional Impairment

A composite variable was used to assess past 30-day functional impairment. The Pre/Post Deployment Study surveys contained a modified Sheehan Disability Scale,^41^ which comprised 4 items that were rated on a 10-point scale (with 0 indicating no interference and 10 indicating very severe). Participants rated the extent to which the problems with their physical health, mental health, or alcohol or drug use were interfering with their home, work, and social lives and close relationships. The composite variable was dummy coded to reflect the endorsement of at least moderate interference on any of the 4 survey items.

#### Psychiatric Disorders

Survey items that were adapted from the Composite International Diagnostic Interview Screening Scales^42^ were used to derive predeployment lifetime and postdeployment past-30-day mental disorder diagnoses.^43^ Assessment of lifetime PTSD in the predeployment survey and of PTSD within the past 30 days in the postdeployment surveys (2 to 3 and 8 to 9 months postdeployment) was based on items from the civilian PTSD Checklist for *DSM-IV* (*Diagnostic and Statistical Manual of Mental Disorders* [Fourth Edition])^44^ and PTSD Checklist for *DSM-5*.^45^ Survey-based mental disorder diagnoses were validated against structured clinical interviews in a previous study.^43^ To adjust for the risk factor of predeployment psychiatric history, binary (yes or no) lifetime internalizing and externalizing disorder composite variables were created. Internalizing disorders included PTSD, MDE, generalized anxiety disorder, panic disorder, or mania or hypomania. Externalizing disorders included substance use disorder, conduct disorder, or oppositional defiant disorder.

#### Responsibility for the Death of Others in Combat

Survey items were taken from the combat stress scale and included “have direct responsibility for the death of” an enemy combatant, a noncombatant, or an ally or US personnel. A binary (≥1 time or never) composite variable was created to indicate any endorsement of responsibility for the death of others in combat. For sensitivity analyses, binary variables were created per type of responsibility (ie, enemy combatant, noncombatant, or ally) and ordinal variables were created by frequency (never, 1 time, or ≥2 times) for any responsibility and by type of responsibility. Variables were created for both predeployment (or previous deployment) and postdeployment (or index deployment) time points.

#### Suicidality

Composite variables were created to assess lifetime (yes or no) and past-30-day (yes or no) STBs, combining the endorsement of suicidal ideation, nonsuicidal self-injury, and suicide attempt. The STB items were evaluated using a self-report version of the Columbia-Suicide Severity Rating Scale.^46^

### Statistical Analysis

Missing data were treated as missing (observations deleted), except for missing combat stress and responsibility for death items, which were imputed as 0. Few participants (22 of 4645 [0.5%]) were missing combat stress and responsibility for death item-level scores, and no participant was missing all responsibility for death or combat stress items. Combined analysis weights were applied in all analyses; these included propensity-based adjustment for baseline attrition (because of incomplete surveys or inability to link to administrative records), poststratification to map the eligible baseline sample to known demographic and service characteristics of soldiers in the 3 BCTs that deployed to Afghanistan after the predeployment interview dates, and propensity-based attrition adjustment to account for the loss of respondents owing to incomplete data in 1 or more follow-up survey waves.

Statistical analyses were conducted using R software, version 3.6.2 (R Foundation for Statistical Computing).^47^ Primary analyses were a series of multivariable logistic regressions that were performed separately for the outcomes at 2 to 3 months and at 8 to 9 months postdeployment. Four outcomes were examined: past 30-day PTSD, MDE, STBs, and functional impairment. Models were adjusted for potential risk factors, including age, sex, race and ethnicity (Black or African American, Hispanic, non-Hispanic White, or other [Asian, American Indian or Alaska Native, Native Hawaiian or Other Pacific Islander, or other], which was self-reported in the survey), marital status, BCT, predeployment lifetime internalizing and externalizing disorders, and combat stress severity (minus responsibility for death items) during the index deployment. Hypothesis tests were 2-sided with a priori *P* < .05 significance level. Data analysis was performed from December 12, 2020, to April 23, 2021.

A series of sensitivity analyses were also conducted that repeated the multivariable logistic regressions: (1) using only responsibility for the death of an enemy combatant, first as a binary variable (yes or no) and then as an ordinal variable (0 = 0 times, 1 = 1 time, or 2 = ≥2 times); (2) using all 3 types of responsibility (enemy combatant, noncombatant, or ally) variables (binary); and (3) including a binary endorsement of responsibility for death during a previous deployment, the index deployment, and their interaction, to assess the potential cumulative effect within the subsample of soldiers who had previously deployed. In addition, given that the study was underpowered to test for specific STBs in regression analyses, we performed Fisher exact tests to examine the bivariate associations between suicidal ideation, nonsuicidal self-injury, and suicide attempt at 2 to 3 vs 8 to 9 months postdeployment and each binary type of responsibility (enemy combatant, noncombatant, ally, or any responsibility). Sensitivity analysis findings are shown in eTables 2 to 4 in the Supplement.

## Results

### Predeployment and Postdeployment Characteristics

A total of 4645 active-duty US Army personnel with data at all 4 survey waves were included in this study. Of this sample, 4358 (94.0%) were male and 276 (6.0%) were female soldiers, with a mean (SD) age of 26.27 (6.07) years; 3032 (65.3%) self-identified as non-Hispanic White, and 3345 (72.4%) reported having a high school education (Table 1). At predeployment, lifetime prevalences were 11.3% (525) for PTSD, 9.1% (425) for MDE, 14.0% (651) for STBs, and 27.7% (1285) for functional impairment in the past 30 days.

**Table 1.** Sociodemographic, Army Service, and Mental Health Characteristics of the Study Sample

Variable	No. (%)			*P* value^a^
	Full sample (n = 4645)	Group with reported responsibility for death (n = 1057)	Group with no reported responsibility for death (n = 3588)	
Age, mean (SD), y	26.27 (6.07)	25.86 (5.54)	26.40 (6.21)	.007
Sex				
Male	4358 (94.0)	1045 (98.8)	3313 (92.3)	<.001
Female	276 (6.0)	10 (1.2)	266 (7.7)	
Race and ethnicity				
Black or African American	467 (10.1)	48 (4.5)	419 (11.7)	<.001
Hispanic	762 (16.4)	135 (12.8)	627 (17.5)	
Non-Hispanic White	3032 (65.3)	800 (75.7)	2232(62.2)	
Other^b^	384 (8.3)	310 (29.3)	310 (8.6)	
Brigade combat team^c^				
A	1454 (31.3)	382 (36.1)	1072 (29.9)	<.001
B	1480 (31.9)	374 (35.4)	1106 (30.8)	
C	1711 (36.8)	201 (19.0)	1410 (39.3)	
Educational level				
GED certificate	289 (6.3)	81 (7.7)	208 (5.8)	<.001
High school diploma	3345 (72.4)	711 (67.3)	2634 (73.4)	
College degree	987 (21.4)	262 (24.8)	725 (20.2)	
Marital status				
Married	2561 (55.4)	585 (55.3)	1966 (54.8)	.75
Never married	1684 (36.5)	376 (35.6)	1308 (36.5)	
Other^d^	375 (8.1)	83 (7.9)	292 (8.1)	
MOS				
Combat arms	1835 (39.5)	579 (54.8)	1256 (35.0)	<.001
Combat support	663 (14.3)	48 (4.5)	615 (17.1)	
Other support	2146 (46.2)	430 (40.7)	1716 (47.8)	
Lifetime predeployment				
PTSD	525 (11.3)	131 (12.4)	394 (11.0)	.20
MDE	425 (9.1)	108 (10.2)	317 (8.8)	.18
STBs	651 (14.0)	148 (14.0)	503 (14.0)	>.99
Suicidal ideation	526 (11.3)	124 (11.7)	402 (11.2)	.66
Nonsuicidal self-injury	256 (5.5)	57 (5.4)	199 (5.5)	.88
Suicide attempt	77 (1.7)	25 (2.4)	52 (1.4)	.05
Internalizing disorder	919 (19.8)	232 (21.9)	687 (19.1)	.05
Externalizing disorder	1252 (26.9)	321 (30.4)	931(25.9)	.01
Predeployment past 30 d				
Functional impairment	1285 (27.7)	310 (29.3)	975 (27.2)	.17
Combat stress score, mean (SD)				
Total (minus responsibility items)	2.54 (1.70)	3.90 (1.67)	2.14 (1.49)	<.001

Abbreviations: GED, General Educational Development; MDE, major depressive episode; MOS, military occupational specialty; PTSD, posttraumatic stress disorder; STBs, suicidal thoughts and behaviors.

^a^ *P* values were calculated using Fisher exact test.

^b^ Race and ethnicity were self-reported in the survey, and other category included Asian, American Indian or Alaska Native, Native Hawaiian or Other Pacific Islander, or other.

^c^ Brigade combat teams were deidentified.

^d^ Other marital status included divorced, separated, and widowed.

The mean (SD) combat stress score during the index deployment was 2.54 (1.70). Correlation strength among combat stress items ranged from small to moderate, with no evidence of multicollinearity (eTable 1 in the Supplement). At 2 to 3 months postdeployment, prevalences within the past 30 days were 7.7% (357) for PTSD, 7.1% (330) for MDE, 3.4% (156) for STBs, and 32.2% (1495) for functional impairment. At 8 to 9 months postdeployment, prevalences within the past 30 days were 11.8% (547) for PTSD, 6.8% (314) for MDE, 6.6% (306) for STBs, and 29.9% (1388) for functional impairment.

### Responsibility for the Death of Others

At the predeployment survey, 20.2% of soldiers (937) reported being responsible for the death of others during a previous deployment (ie, 40.8% [937 of 2299] of those who deployed previously) (Table 2). Of those 937 soldiers, 90.3% (846) reported being responsible for the death of an enemy combatant, 31.4% (294) reported being responsible for the death of a noncombatant, and 4.4% (41) reported being responsible for the death of an ally.

**Table 2.** Reported Responsibility for the Death of Others per Deployment by Type and Frequency^a^

Variable	No./total No. of US soldiers (%)
**Previous deployment (n = 2299)**	
Responsibility for death	
Any	937/2299 (40.8)
Enemy combatant	846/937 (90.3)
Noncombatant	294/937 (31.4)
US personnel or ally	41/937 (4.4)
Frequency	
Any 1 time	246/2299 (10.7)
Any ≥2 times	691/2299 (30.1)
Enemy combatant 1 time	191/2299 (8.3)
Enemy combatant ≥2 times	655/2299 (28.5)
Noncombatant 1 time	196/2299 (8.5)
Noncombatant ≥2 times	98/2299 (4.3)
Ally 1 time	23/2299 (1.0)
Ally ≥2 times	18/2299 (0.8)
**Index deployment (n = 4645)**	
Responsibility for death	
Any	1057/4645 (22.8)
Enemy combatant	1025/1057 (97.0)
Noncombatant	80/1057 (7.6)
US personnel or ally	44/1057 (4.2)
Frequency	
Any 1 time	363/4645 (7.8)
Any ≥2 times	694/4645 (14.9)
Enemy combatant 1 time	361/4645 (7.8)
Enemy combatant ≥2 times	664/4645 (14.3)
Noncombatant 1 time	48/4645 (1.0)
Noncombatant ≥2 times	32/4645 (0.7)
Ally 1 time	33/4645 (0.7)
Ally ≥2 times	11/4645 (0.2)

^a^ Categories are not mutually exclusive.

After returning from Afghanistan, 22.8% of soldiers (1057 of 4645) reported being responsible for the death of another during the index deployment. These soldiers were more likely to self-identify as non-Hispanic White, be serving in combat arms roles, and have previous internalizing and externalizing disorders (Table 1). Of those 1057 soldiers, 97.0% (1025) reported being responsible for the death of an enemy combatant, 7.6% (80) reported being responsible for the death of a noncombatant, and 4.2% (44) reported being responsible for the death of an ally. Notably, 77.5% (62) of the 80 soldiers who were responsible for a noncombatant death and 61.4% (27) of the 44 soldiers who were responsible for an ally death were also responsible for an enemy combatant death (Table 2). Approximately 36% of respondents (380 of 1057) reported responsibility for the death of others during both past and current deployments.

### Primary Analyses

When adjusting for sex, age, race and ethnicity, marital status, educational level, BCT, predeployment lifetime internalizing and externalizing disorders, and combat stress, we found that being responsible for the death of others during combat was not associated with any outcomes at 2 to 3 months postdeployment (PTSD odds ratio [OR]: 1.23 [95% CI, 0.93-1.63]; *P* = .14; STB OR: 1.19 [95% CI, 0.84-1.68]; *P* = .33; MDE OR: 1.03 [95% CI, 0.73-1.45]; *P* = .87; and functional impairment OR: 1.12 [95% CI, 0.94-1.34]; *P* = .19) (Table 3). However, being responsible for the death of others was significantly associated with PTSD (OR, 1.42; 95% CI, 1.09-1.86; *P* = .01) and STBs (OR, 1.55; 95% CI, 1.03-2.33; *P* = .04) at 8 to 9 months postdeployment. Being responsible was not associated with MDE (OR, 1.30; 95% CI, 0.93-1.81; *P* = .13) or functional impairment (OR, 1.13; 95% CI, 0.94-1.36; *P* = .19) at 8 to 9 months postdeployment (Table 3).

**Table 3.** Multivariable Logistic Regression Analysis of Postdeployment Outcomes

Variable	2-3 mo Postdeployment		8-9 mo Postdeployment	
	OR (95% CI)	*P* value	OR (95% CI)	*P* value
**PTSD**				
Age	1.02 (0.10-1.05)	.11	1.01 (0.10-1.03)	.11
Female sex	1.61 (0.97-2.67)	.07	2.14 (1.42-3.22)	<.001
Male sex	1 [Reference]		1 [Reference]	
Race and ethnicity				
Black or African American	0.88 (0.52-1.51)	.04	1.14 (0.74-1.77)	.008
Hispanic	1.40 (0.99-1.97)		1.32 (1.04-1.68)	
Non-Hispanic White	1 [Reference]		1 [Reference]	
Other^a^	1.67 (1.08-2.58)		1.53 (1.04-2.25)	
Brigade combat team				
A	1 [Reference]	.04	1 [Reference]	.34
B	1.47 (1.07-2.01)		0.90 (0.71-1.16)	
C	1.09 (0.79-1.51)		1.01 (0.85-1.32)	
Educational level				
No high school diploma	1 [Reference]	.43	1 [Reference]	<.001
High school diploma	0.83 (0.54-1.26)		0.62 (0.42-0.91)	
College degree	0.70 (0.40-1.21)		0.41 (0.27-0.63)	
Marital status				
Married	1 [Reference]	.42	1 [Reference]	.21
Never married	0.84 (0.59-1.21)		0.84 (0.67-1.07)	
Other^b^	1.15 (0.89-1.69)		1.14 (0.96-1.52)	
Internalizing disorder	4.31 (3.35-5.54)	<.001	3.69 (2.90-4.70)	<.001
Externalizing disorder	1.45 (1.12-1.86)	.003	1.33 (1.10-1.61)	.003
Combat severity	1.42 (1.30-1.55)	<.001	1.26 (1.20-1.34)	<.001
Responsible for death	1.23 (0.93-1.63)	.14	1.42 (1.09-1.86)	.01
**STBs**				
Age	0.97 (0.93-1.01)	.16	1.01 (0.99-1.04)	.43
Female sex	1.69 (0.88-3.24)	.11	2.03 (1.14-3.62)	.02
Male sex	1 [Reference]		1 [Reference]	
Race and ethnicity				
Black or African American	0.86 (0.48-1.55)	.95	1.09 (0.68-1.75)	.36
Hispanic	1.09 (0.63-1.89)		1.34 (0.90-2.01)	
Non-Hispanic White	1 [Reference]		1 [Reference]	
Other^a^	1.04 (0.51-2.14)		1.42 (0.85-2.38)	
Brigade combat team				
A	1 [Reference]	.38	1 [Reference]	.25
B	1.24 (0.90-1.71)		0.88 (0.59-1.31)	
C	1.10 (0.76-1.58)		1.21 (0.87-1.69)	
Educational level				
No high school diploma	1 [Reference]	.13	1 [Reference]	.58
High school diploma	1.49 (0.69-3.22)		0.88 (0.61-1.21)	
College degree	1.84 (0.92-3.67)		0.82 (0.55-1.21)	
Marital status				
Married	1 [Reference]	.04	1 [Reference]	.09
Never married	1.41 (0.96-2.08)		1.38 (0.96-1.98)	
Other^b^	1.81 (1.09-2.99)		1.43 (0.93-2.19)	
Internalizing disorder	3.15 (2.15-4.62)	<.001	2.60 (1.96-3.46)	<.001
Externalizing disorder	1.62 (1.14-2.31)	.008	1.93 (1.42-2.61)	<.001
Combat severity	1.02 (0.92-1.12)	.74	1.03 (0.96-1.11)	.43
Responsible for death	1.19 (0.84-1.68)	.33	1.55 (1.03-2.33)	.04
**MDE**				
Age	1.00 (0.97-1.04)	.96	1.02 (0.10-1.04)	.08
Female sex	1.65 (0.98-2.76)	.06	2.03 (1.10-3.75)	.02
Male sex	1 [Reference]		1 [Reference]	
Race and ethnicity				
Black or African American	1.39 (0.93-2.06)	.02	1.22 (0.78-1.91)	.28
Hispanic	0.96 (0.63-1.44)		1.15 (0.76-1.74)	
Non-Hispanic White	1 [Reference]		1 [Reference]	
Other^a^	1.69 (1.12-2.56)		1.42 (0.95-2.13)	
Brigade combat team				
A	1 [Reference]	.06	1 [Reference]	.06
B	1.39 (1.03-1.87)		0.88 (0.61-1.26)	
C	1.14 (0.84-1.56)		1.20 (0.93-1.55)	
Educational level				
No high school diploma	1 [Reference]	.56	1 [Reference]	.002
High school diploma	0.97 (0.68-1.39)		0.72 (0.48-1.07)	
College degree	0.82 (0.54-1.26)		0.44 (0.26-0.72)	
Marital status				
Married	1 [Reference]	.62	1 [Reference]	.22
Never married	0.90 (0.64-1.26)		0.96 (0.69-1.33)	
Other^b^	0.82 (0.51-1.29)		1.38 (0.93-2.04)	
Internalizing disorder	5.87 (4.34-7.94)	<.001	3.67 (2.75-4.90)	<.001
Externalizing disorder	1.78 (1.35-2.34)	<.001	1.31 (1.02-1.67)	.03
Combat severity	1.20 (1.09-1.32)	<.001	1.15 (1.06-1.25)	.001
Responsible for death	1.03 (0.73-1.45)	.87	1.30 (0.93-1.81)	.13
**Functional impairment**				
Age	1.00 (0.98-1.01)	.44	1.01 (0.99-1.02)	.30
Female sex	1.02 (0.69-1.51)	.93	1.78 (1.26-2.51)	.001
Male sex	1 [Reference]		1 [Reference]	
Race and ethnicity				
Black or African American	1.47 (0.92-1.43)	.002	1.17 (0.91-1.50)	.009
Hispanic	1.33 (1.10-1.61)		1.31 (1.08-1.64)	
Non-Hispanic White	1 [Reference]		1 [Reference]	
Other^a^	1.47 (1.14-1.90)		1.27 (0.99-1.64)	
Brigade combat team				
A	1 [Reference]	.44	1 [Reference]	.001
B	1.12 (0.92-1.39)		1.10 (0.88-1.36)	
C	1.10 (0.91-1.34)		1.43 (1.18-1.72)	
Educational level				
No high school diploma	1 [Reference]	.60	1 [Reference]	<.001
High school diploma	0.93 (0.69-1.26)		0.69 (0.52-0.92)	
College degree	0.87 (0.63-1.21)		0.48 (0.35-0.68)	
Marital status				
Married	1 [Reference]	.02	1 [Reference]	.07
Never married	0.98 (0.83-1.14)		1.14 (0.99-1.31)	
Other^b^	1.42 (1.11-1.80)		1.21 (0.92-1.59)	
Internalizing disorder	2.65 (2.21-3.17)	<.001	2.67 (2.24-3.17)	<.001
Externalizing disorder	1.38 (1.16-1.65)	<.001	1.42 (1.25-1.62)	<.001
Combat severity	1.12 (1.07-1.18)	<.001	1.13 (1.08-1.19)	<.001
Responsible for death	1.12 (0.94-1.34)	.19	1.13 (0.94-1.36)	.19

Abbreviations: MDE, major depressive episode; OR, odds ratio; PTSD, posttraumatic stress disorder; STBs, suicidal thoughts and behaviors.

^a^ Race and ethnicity were self-reported in the survey, and other category included Asian, American Indian or Alaska Native, Native Hawaiian or Other Pacific Islander, or other.

^b^ Other marital status included divorced, separated, and widowed.

### Sensitivity Analyses

Repeating the adjusted regressions by entering into the models only the responsibility for the death of an enemy combatant (binary) showed that being responsible remained significantly associated with PTSD at 8 to 9 months postdeployment (OR, 1.44; 95 CI%, 1.10-1.90; *P* = .009) (Table 4). However, the association with STBs at 8 to 9 months postdeployment was attenuated (OR, 1.46; 95 CI%, 0.97- 2.20; *P* = .07). Patterns of other findings were unchanged. Regressions that were repeated using an ordinal operationalization showed that being responsible for an enemy combatant’s death only once (OR, 1.58; 95 CI%, 1.16-2.16; *P* = .01) or multiple times (OR, 1.36; 95 CI%, 1.02-1.82; *P* = .01) was associated with higher odds of PTSD at 8 to 9 months postdeployment (Table 5).

**Table 4.** Logistic Regression Analysis With Only Responsibility for the Death of Enemy Combatants^a^

Variable	2-3 mo Postdeployment		8-9 mo Postdeployment	
	OR (95% CI)	*P* value	OR (95% CI)	*P* value
PTSD				
Internalizing disorder	4.31 (3.35-5.54)	<.001	3.69 (2.90-4.70)	<.001
Externalizing disorder	1.45 (1.13-1.86)	.003	1.33 (1.00-1.61)	.003
Combat severity	1.42 (1.30-1.55)	<.001	1.26 (1.19-1.34)	<.001
Enemy combatant	1.25 (0.93-1.69)	.13	1.44 (1.10-1.90)	.009
STBs				
Internalizing disorder	3.15 (2.15-4.62)	<.001	2.60 (1.96-3.46)	<.001
Externalizing disorder	1.62 (1.14-2.32)	.007	1.94 (1.43-2.61)	<.001
Combat severity	1.03 (0.93-1.13)	.59	1.04 (0.96-1.12)	.32
Enemy combatant	1.09 (0.77-1.54)	.62	1.46 (0.97-2.20)	.07
MDE				
Internalizing disorder	5.87 (4.33-7.94)	<.001	3.68 (2.76-4.90)	<.001
Externalizing disorder	1.77 (1.35-2.34)	<.001	1.31 (1.02-1.67)	.03
Combat severity	1.19 (1.19-1.08)	<.001	1.15 (1.06-1.25)	.001
Enemy combatant	1.10 (0.78-1.56)	.58	1.33 (0.96-1.85)	.10
Functional impairment				
Internalizing disorder	2.65 (2.21-3.17)	<.001	2.61 (2.20-3.10)	<.001
Externalizing disorder	1.38 (1.16-1.65)	<.001	1.42 (1.25-1.62)	<.001
Combat severity	1.13 (1.07-1.18)	<.001	1.14 (1.09-1.20)	<.001
Enemy combatant	1.11 (0.92-1.33)	.28	1.11 (0.92-1.34)	.29

Abbreviations: MDE, major depressive episode; OR, odds ratio; PTSD, posttraumatic stress disorder; STBs, suicidal thoughts and behaviors.

^a^ Models were adjusted for age, sex, race and ethnicity, educational level, brigade combat team, and marital status. Internalizing and externalizing disorders were lifetime and reported at the predeployment survey.

**Table 5.** Logistic Regression Analysis With Frequency of Responsibility for the Death of Enemy Combatants^a^

Variable	2-3 mo Postdeployment		8-9 mo Postdeployment	
	OR (95% CI)	*P* value	OR (95% CI)	*P* value
**PTSD**				
Internalizing disorder	4.29 (3.33-5.52)	<.001	3.70 (2.90-4.73)	<.001
Externalizing disorder	1.46 (1.14-1.87)	.003	1.33 (1.10-1.60)	.003
Combat severity	1.41 (1.29-1.54)	<.001	1.27 (1.19-1.34)	<.001
Enemy combatant				
1 Time	1.01 (0.64-1.58)	.11	1.58 (1.16-2.16)	.01
≥2 Times	1.40 (1.01-1.94)		1.36 (1.02-1.82)	
Never	1 [Reference]		1 [Reference]	
**STBs**				
Internalizing disorder	3.15 (2.15-4.61)	<.001	2.60 (1.96-3.45)	<.001
Externalizing disorder	1.63 (1.14-2.32)	.007	1.94 (1.43-2.62)	<.001
Combat severity	1.03 (0.93-1.13)	.61	1.04 (0.96-3.45)	.38
Enemy combatant				
1 Time	1.03 (0.62-1.72)	.87	1.27 (0.81-1.20)	.17
≥2 Times	1.13 (0.71-1.80)		1.58 (0.98-2.54)	
Never	1 [Reference]		1 [Reference]	
**MDE**				
Internalizing disorder	5.90 (4.35-8.02)	<.001	3.68 (2.76-4.91)	<.001
Externalizing disorder	1.78 (1.34-2.33)	<.001	1.30 (1.02-1.67)	.03
Combat severity	1.20 (1.09-1.32)	<.001	1.15 (1.05-1.25)	.002
Enemy combatant				
1 Time	1.35 (0.88-2.06)	.32	1.37 (0.90-2.08)	.23
≥2 Times	0.97 (0.64-1.47)		1.31 (0.90-1.90)	
Never	1 [Reference]		1 [Reference]	
**Functional impairment**				
Internalizing disorder	2.65 (2.21-3.18)	<.001	2.67 (2.24-3.17)	<.001
Externalizing disorder	1.38 (1.16-1.64)	<.001	1.42 (1.25-1.62)	<.001
Combat severity	1.13 (1.07-1.19)	<.001	1.14 (1.09-1.19)	<.001
Enemy combatant				
1 Time	1.24 (0.96-1.62)	.26	1.11 (0.91-1.36)	.51
≥2 Times	1.03 (0.82-1.30)		1.10 (0.87-1.39)	
Never	1 [Reference]		1 [Reference]	

Abbreviations: MDE, major depressive episode; OR, odds ratio; PTSD, posttraumatic stress disorder; STBs, suicidal thoughts and behaviors.

^a^ Models were adjusted for age, sex, race and ethnicity, educational level, brigade combat team, and marital status. Internalizing and externalizing disorders were lifetime and reported at the predeployment survey.

In adjusted regressions that simultaneously included in the models all 3 types of responsibility (eTable 3 in the Supplement), responsibility for the death of an enemy combatant remained significantly associated with PTSD (OR, 1.49; 95 CI%, 1.12-1.98; *P* = .007) but not with STBs (OR, 1.47; 95 CI%, 0.96-2.26; *P* = .08) at 8 to 9 months postdeployment. Neither of the other types of responsibility was significantly associated with PTSD, MDE, STBs, or functional impairment. Analyses were repeated in the subsample of soldiers who had previously deployed (n = 2228). No evidence was found of a cumulative effect of responsibility for the death of others during multiple deployments (eTable 4 in the Supplement).

## Discussion

In a sample of post–September 11, 2001 active-duty soldiers who were deployed to Afghanistan, those who reported responsibility for the death of others were 42% (OR, 1.42) more likely to have PTSD and were 55% (OR, 1.55) more likely to have STBs at 8 to 9 months postdeployment. These associations were not significant at 2 to 3 months postdeployment, suggesting the possibility of a critical window for intervention. Being responsible for the death of others was not associated with MDE or functional impairment at any time after deployment.

These findings replicate and build on the evidence in veterans from multiple war eras that demonstrated an association between being responsible for another’s death during combat and subsequent PTSD and STBs.^17,18,21,26,27,28,29^ We found that these associations are detectable while soldiers are still on active duty, which suggests that an opportunity exists for screening and intervention, potentially disrupting the often chronic, treatment-refractory trajectories in veterans with military-related PTSD.^48^ In addition, these findings identify the psychological sequelae of being responsible for another’s death as a potential intervention target for PTSD psychotherapy at 8 to 9 months postdeployment. The pattern remained the same for PTSD when examining specifically the responsibility for an enemy combatant’s death, although the association with STB was attenuated.

These findings contrast with those of a study of 400 soldiers that found that unjust war events (but not killing enemy combatants) were associated with adverse mental health outcomes.^31^ However, operationalization differences may at least partially explain the discrepancies. Specifically, in that study, “unjust war events” included being responsible for noncombatant deaths (as well as witnessing brutality toward noncombatants, ill or injured women or children, and Geneva Convention violations), and “killing enemy combatants” included an item that can be categorized as a fighting variable (“shooting/directing fire at enemy”).^49^ These alternative ways of categorizing combat-related events, therefore, do not address directly the question assessed here. Although multivariable logistic regression models did not show associations between being responsible for the deaths of noncombatants or allies and PTSD or STBs, the study was underpowered to detect such an association, and those results should be interpreted cautiously.

Shifting away from a primarily fear-based trauma framework, we believe that the current findings shed light on the association between active participation in death in combat and mental health. Moving forward, research should examine the predictive value of expanding the conceptualizations of criterion A of the *DSM-5* PTSD diagnosis to include such events. Responsibility for the death of others during combat may confer unique cognitive or affective mechanisms (eg, shame and guilt) that contribute to a more complex pattern of PTSD.^24^ Similarly, suicidality at 8 to 9 months after being responsible for another’s death may reflect a costly attempt at making amends or the consequence of separating from a unit that provided meaning-making from the experience.^50^ Future investigation into soldiers’ perceptions of moral transgressions and moral injury^51,52^ will help clarify the basis for the associations found in the current study, thus identifying actionable intervention targets.^53,54,55^ A broader perspective of PTSD mechanisms beyond fear will be important in identifying possible subtypes of the disorder, additional neurobiological mechanisms, and precision treatment needs.

These alternative conceptualizations may be a possible explanation for the low success rates of first-line psychotherapy approaches for military-related PTSD in active-duty soldiers, with only 31% of participants achieving recovery.^56^ However, 2 psychotherapy approaches (Adaptive Disclosure^57^ and Impact of Killing in War^58^) that address the psychological sequelae of killing in combat show promising preliminary results in reducing PTSD in active-duty Marines and sailors and veterans, respectively. These treatment options may benefit soldiers with postdeployment PTSD who reported responsibility for the death of others, but additional research is needed. It is unknown whether current first-line psychotherapy is useful for PTSD that is associated with being responsible for another’s death. It is critical to ascertain whether therapeutically addressing such topics while soldiers remain in service, with the possibility of future combat deployment, is beneficial or iatrogenic. Until then, a possible primary prevention strategy may be for units to prepare soldiers before deployment by discussing the possibility and impact of being responsible for another’s death in combat (including fratricide and noncombatants).

### Limitations

This study has several limitations. First, symptom measures and combat experiences were assessed with self-reported questionnaires,^59^ which are subject to response biases, including social desirability. Responsibility for the death of others was as reported by the soldier not as ascertained or adjudicated by any military or other source. Respondents may have endorsed being responsible across a range of conditions (eg, active killing, giving orders, legal responsibility, and feeling responsible), and future studies should identify whether these differences have meaningful implications for the associations with mental health symptoms. Second, these findings in a sample of US Army personnel who were deployed to Afghanistan in 2012 may not be generalizable to other periods or conflicts. The sample was restricted to soldiers with data at all 4 survey waves, which may have altered the results, although weights were used to mitigate the impacts of nonparticipation and attrition. Third, because of low endorsements, the study was underpowered to test for differential associations of ally and noncombatant deaths with postdeployment mental health outcomes. Similarly, the study was underpowered to examine specific STB outcomes (eg, suicidal ideation and suicide attempts), which demonstrate distinct associations with risk factors.^60^ Future research should focus on the association between these specific combat experiences and suicide attempt and death by suicide, separately. Fourth, future studies should examine common pathways and additive risk models of PTSD and STBs.

## Conclusions

This cohort study found that being responsible for the death of others during combat is associated with PTSD and STB at 8 to 9 months, but not 2 to 3 months, postdeployment. These findings shed light on complex war traumas and their sequelae. Identifying soldiers who report this type of responsibility on their return from deployment and delivering interventions early may mitigate subsequent PTSD and STBs.

## Notes

Supplement. **eTable 1.** Bivariate Associations of Combat Stress Scale Items From Index Deployment **eTable 2.** Bivariate Associations of Specific Post-Deployment STBs and Responsibility Type **eTable 3.** Logistic Regression With All Types of Responsibilities Predicting Post-Deployment Outcomes **eTable 4.** Logistic Regression With Prior and Index Responsibilities Predicting Post-Deployment Outcomes Within Only Those Who Deployed Previously
