# Supplementary material for: Association Between Responsibility for the Death of Others and Postdeployment Mental Health and Functioning in US Soldiers
Source: JAMA Netw Open. 2021 Nov 1;4(11):e2130810. doi: 10.1001/jamanetworkopen.2021.30810 (PMC8561325; doi:10.1001/jamanetworkopen.2021.30810)
Supplement: Supplement. — eTable 1. Bivariate Associations of Combat Stress Scale Items From Index Deployment eTable 2. Bivariate Associations of Specific Post-Deployment STBs and Responsibility Type eTable 3. Logistic Regression With All Types of Responsibilities Predicting Post-Deployment Outcomes eTable 4. Logistic Regression With Prior and Index Responsibilities Predicting Post-Deployment Outcomes Within Only Those Who Deployed Previously [file jamanetwopen-e2130810-s001.pdf]

Supplementary Online Content

Khan AJ, Campbell-Sills L, Sun X, et al. Association between responsibility for the death of others and postdeployment mental health and functioning in US soldiers. *JAMA Netw Open*. 2021;4(11):e2130810. doi:10.1001/jamanetworkopen.2021.30810

eTable 1. Bivariate Associations of Combat Stress Scale Items From Index Deployment

eTable 2. Bivariate Associations of Specific Post-Deployment STBs and Responsibility Type

eTable 3. Logistic Regression With All Types of Responsibilities Predicting Post-Deployment Outcomes

eTable 4. Logistic Regression With Prior and Index Responsibilities Predicting Post-Deployment Outcomes Within Only Those Who Deployed Previously

This supplementary material has been provided by the authors to give readers additional information about their work.

© 2021 Khan AJ et al. *JAMA Network Open.*

eTable 1. Bivariate Associations of Combat Stress Scale Items From Index Deployment

	1.	2.	3.	4.	5.	6.	7.	8.	9.	10.
1. Responsible for Enemy Combatant Death	-	.18	.10	.23	.46	.15	.34	.17	.18	.19
2. Responsible for Non-Combatant Death		-	.11	.05	.12	.08	.09	.07	.04	.09
3. Responsible for U.S. or Ally Death			-	**.03**	.04	**.03**	.05	.05	**.02**	.04
4. Combat Patrol/Dangerous Duty				-	.28	.12	.32	.14	.43	.15
5. Fired Rounds/Took Fire					-	.14	.39	.15	.17	.23
6. Personally Wounded						-	.18	.08	.10	.07
7. Had a Close Call							-	.19	.24	.23
8. Unit Member Killed or Wounded								-	.22	.16
9. Saw Villages Destroyed or People Begging for Food									-	.17
10. Exposed to Aftermath (severely wounded, dead bodies)										-

Note. All correlations $P<0.01$ except bolded items which are $P>0.05$. Spearman correlations. Items reflect weighted scores.

© 2021 Khan AJ et al. *JAMA Network Open.*

eTable 2. Bivariate Associations of Specific Post-Deployment STBs and Responsibility Type

	SI, N(%)				NSSI, N(%)				SA, N(%)			
**Type of Death**	T2	P value	T3	P value	T2	P value	T3	P value	T2	P value	T3	P value
Enemy<br>Yes	34(3.3)	0.40	70(6.8)	0.03	8(0.8)	>0.99	20(2.0)	0.09	4(0.4)	0.02	6(0.6)	0.68
No	102(2.8)		181(5)		28(0.8)		44(1.2)		2 (0.1)		27(0.7)	
Non-Combatant<br>Yes	3(3.8)	0.51	5(6.2)	0.62	0(0)	>0.99	1(1.2)	>0.99	0(0)	>0.99	0(0)	>0.99
No	133(2.9)		246(5.4)		36(0.8)		63(1.4)		6(0.1)		33(0.7)	
Ally<br>Yes	4(9.1)	0.04	5(11.4)	0.09	0(0)	>0.99	1(2.3)	0..46	0(0)	>0.99	2(4.5)	0.04
No	132(2.9)		246(5.3)		36(0.8)		63(1.4)		6(0.1)		31(0.7)	
Any Death<br>Yes	37(3.5)	0.21	74(7.0)	0.01	8(0.8)	>0.99	21(2.0)	0.07	4(0.4)	0.03	8(0.8)	0.84
No	99(2.8)		177(4.9)		28(0.8)		43(1.2)		2(0.4)		25(0.7)	

Note. P-value calculated from Fisher's exact test. Percentage reflects suicidality outcome prevalence based on endorsement of being responsible for another's death. T2 = 2-3 months post-deployment; T3 = 8-9 months post-deployment. SI = suicidal ideation; NSSI = non-suicidal self-injury; SA = suicide attempt. All outcomes are past 30-days disorder = Lifetime at pre-deployment baseline

© 2021 Khan AJ et al. *JAMA Network Open.*

eTable 3. Logistic Regression With All Types of Responsibilities Predicting Post-Deployment Outcomes

	Odds Ratio (95% CI)	P value	Odds Ratio (95% CI)	P value
Variable	2-3 Months Post-Deployment		8-9 Months Post-Deployment	
	**Posttraumatic Stress Disorder**			
Internalizing Disorder	4.17 (3.22-5.40)	<0.001	3.58 (2.79-4.59)	<0.001
Externalizing Disorder	1.41 (1.10-1.81)	0.007	1.34 (1.11-1.61)	0.003
Combat Severity	1.43 (1.31-1.57)	<0.001	1.26 (1.19-1.24)	<0.001
Enemy Combatant	1.25 (0.92-1.70)	0.15	1.49 (1.12-1.98)	0.007
Non-Combatant	1.03 (0.49-2.14)	0.95	0.86 (0.51-1.46)	0.57
Ally	1.10 (0.42-2.86)	0.85	0.94 (0.38-2.33)	0.89
	**Suicidal Thoughts and Behaviors**			
Internalizing Disorder	3.06 (2.09-4.50)	<0.001	2.58 (1.94-3.44)	<0.001
Externalizing Disorder	1.58 (1.10-2.27)	0.01	1.93 (1.42-2.63)	<0.001
Combat Severity	1.02 (0.92-1.13)	0.72	1.03 (0.96-1.12)	0.40
Enemy Combatant	1.10 (0.76-1.58)	0.62	1.47 (0.96-2.26)	0.08
Non-Combatant	0.92 (0.25-3.43)	0.90	0.80 (0.25-2.52)	0.70
Ally	2.54 (0.83-7.79)	0.10	1.79 (0.71-4.52)	0.22
	**Major Depressive Episode**			
Internalizing Disorder	5.87 (4.33-7.96)	<0.001	3.61 (2.68-4.86)	<0.001
Externalizing Disorder	1.73 (1.31-2.28)	<0.001	1.25 (0.97-1.61)	0.08
Combat Severity	1.20 (1.09-1.32)	<0.001	1.16 (1.06-1.27)	0.001
Enemy Combatant	1.31 (0.80-1.59)	0.48	1.37 (0.98-1.92)	0.07
Non-Combatant	0.47 (0.18-1.23)	0.12	0.62 (0.19-2.07)	0.44
Ally	0.94 (0.32-2.81)	0.92	0.60 (0.19-1.91)	0.39
	**Functional Impairment**			
Internalizing Disorder	2.60 (2.17-3.12)	<0.001	2.61 (2.20-3.10)	<0.001
Externalizing Disorder	1.39 (1.16-1.67)	<0.001	1.43 (1.25-1.63)	<0.001
Combat Severity	1.12 (1.07-1.18)	<0.001	1.14 (1.09-1.20)	<0.001
Enemy Combatant	1.10 (0.92-1.32)	0.31	1.10 (0.90-1.34)	0.34
Non-Combatant	1.17 (0.65-2.11)	0.61	1.36 (0.86-2.15)	0.19
Ally	1.14 (0.58-2.25)	0.72	0.67 (0.34-1.33)	0.25

Note. Models adjusted for age, sex, race/ethnicity, education, BCT (brigade combat team), and martial status. Internalizing and externalizing = Lifetime at pre-deployment baseline.

© 2021 Khan AJ et al. *JAMA Network Open.*

eTable 4. Logistic Regression With Prior and Index Responsibilities Predicting Post-Deployment Outcomes Within Only Those Who Deployed Previously

	Odds Ratio (95% CI)	P value	Odds Ratio (95% CI)	P value
Variable	2-3 Months Post-Deployment		8-9 Months Post-Deployment	
	**Posttraumatic Stress Disorder**			
Internalizing Disorder	5.42 (3.75-7.82)	<0.001	5.14 (3.81-6.94)	<0.001
Externalizing Disorder	1.36 (0.93-2.01)	0.12	1.22 (0.93-1.62)	0.16
Combat Severity	1.38 (1.25-1.53)	<0.001	1.32 (1.24-1.42)	<0.001
Prior Responsibility	1.19 (0.76-1.86)	0.44	1.22 (0.81-1.85)	0.34
Index Responsibility	1.32 (0.74-2.34)	0.35	1.14 (0.65-2.00)	0.66
Prior*Index Interaction	0.92 (0.49-1.73)	0.79	1.08 (0.52-2.25)	0.83
	**Suicidal Thoughts and Behaviors**			
Internalizing Disorder	4.12 (2.40-7.09)	<0.001	2.88 (2.07-4.01)	<0.001
Externalizing Disorder	2.04 (1.14-3.62)	0.02	2.08 (1.36-3.19)	0.001
Combat Severity	0.96 (0.79-1.16)	0.66	1.11 (1.00-1.23)	0.06
Prior Responsibility	1.03 (0.55-1.91)	0.94	1.04 (0.63-1.70)	0.89
Index Responsibility	0.89 (0.24-2.61)	0.70	1.01 (0.44-2.34)	0.98
Prior*Index Interaction	2.04 (0.50-8.25)	0.32	1.64 (0.62-4.30)	0.32
	**Major Depressive Episode**			
Internalizing Disorder	6.76(4.26-10.72)	<0.001	3.90 (2.67-5.69)	<0.001
Externalizing Disorder	1.74 (1.23-2.47)	0.002	1.51 (1.03-2.24)	0.03
Combat Severity	1.21 (1.06-1.37)	0.004	1.19 (1.05-1.35)	0.006
Prior Responsibility	1.09 (0.64-1.86)	0.75	1.44 (0.88-2.35)	0.15
Index Responsibility	0.95 (0.49-1.85)	0.88	0.62 (0.26-1.48)	0.28
Prior*Index Interaction	1.12 (0.53-2.39)	0.76	2.46 (0.93-6.48)	0.07
	**Functional Impairment**			
Internalizing Disorder	2.63 (2.06-3.34)	<0.001	2.96 (2.35-3.74)	<0.001
Externalizing Disorder	1.31 (1.04-1.66)	0.02	1.36 (1.12-1.66)	0.002
Combat Severity	1.15 (1.07-1.23)	<0.001	1.20 (1.11-1.28)	<0.001
Prior Responsibility	0.89 (0.68-1.17)	0.41	0.96 (0.76-1.20)	0.71
Index Responsibility	1.04 (0.73-1.48)	0.82	1.12 (0.73-1.66)	0.52
Prior*Index Interaction	1.04 (0.67-1.62)	0.87	1.10 (0.69-1.75)	0.70

Note. Models adjusted for age, sex, race/ethnicity, education, BCT (brigade combat team), and martial status. Internalizing and externalizing = Lifetime at pre-deployment baseline. Responsibility = binary endorsement of being responsible for another’s death during combat during prior and index deployments.

© 2021 Khan AJ et al. *JAMA Network Open.*
